# Mesenchymal-Derived Extracellular Vesicles Enhance Microglia-mediated Synapse Remodeling after Cortical Injury in Rhesus Monkeys

**DOI:** 10.21203/rs.3.rs-2917340/v1

**Published:** 2023-05-15

**Authors:** Yuxin Zhou, Hrishti Bhatt, Chromewell A. Mojica, Hongqi Xin, Monica Pessina, Douglas L. Rosene, Tara L. Moore, Maria Medalla

**Affiliations:** Boston University Chobanian & Avedisian School of Medicine; Boston University Chobanian & Avedisian School of Medicine; Boston University Chobanian & Avedisian School of Medicine; Henry Ford Health Systems; Boston University Chobanian & Avedisian School of Medicine; Boston University Chobanian & Avedisian School of Medicine; Boston University Chobanian & Avedisian School of Medicine; Boston University Chobanian & Avedisian School of Medicine

**Keywords:** cortical injury, synaptic plasticity, neuroinflammation, extracellular vesicles, microglia, C1q

## Abstract

Understanding the microglial neuro-immune interactions in the primate brain is vital to developing therapeutics for cortical injury, such as stroke. Our previous work showed that mesenchymal-derived extracellular vesicles (MSC-EVs) enhanced motor recovery in aged rhesus monkeys post-injury of primary motor cortex (M1), by promoting homeostatic ramified microglia, reducing injury-related neuronal hyperexcitability, and enhancing synaptic plasticity in perilesional cortices. The current study addresses how these injury- and recovery-associated changes relate to structural and molecular interactions between microglia and neuronal synapses. Using multi-labeling immunohistochemistry, high resolution microscopy, and gene expression analysis, we quantified co-expression of synaptic markers (VGLUTs, GLURs, VGAT, GABARs), microglia markers (Iba-1, P2RY12), and C1q, a complement pathway protein for microglia-mediated synapse phagocytosis, in perilesional M1 and premotor cortices (PMC) of monkeys with intravenous infusions of either vehicle (veh) or EVs post-injury. We compared this lesion cohort to aged-matched non-lesion controls. Our findings revealed a lesion-related loss of excitatory synapses in perilesional areas, which was ameliorated by EV treatment. Further, we found region-dependent effects of EV on microglia and C1q expression. In perilesional M1, EV treatment and enhanced functional recovery were associated with increased expression of C1q + hypertrophic microglia, which are thought to have a role in debris-clearance and anti-inflammatory functions. In PMC, EV treatment was associated with decreased C1q + synaptic tagging and microglial-spine contacts. Our results provided evidence that EV treatment facilitated synaptic plasticity by enhancing clearance of acute damage in perilesional M1, and thereby preventing chronic inflammation and excessive synaptic loss in PMC. These mechanisms may act to preserve synaptic cortical motor networks and a balanced normative M1/PMC synaptic connectivity to support functional recovery after injury.

## 1. INTRODUCTION

Cortical injury, such as stroke in humans, causes neuronal damage and loss of synaptic connections, leading to significant cognitive and behavioral impairments [1, 2]. Brain plasticity enables functional recovery after cortical injury, which is modulated by neuro-inflammatory responses [3]. As the immune cells of the brain, microglia can promote neuronal plasticity and recovery via phagocytosis of damaged pre- and post-synaptic elements, and release of neurotrophic factors to facilitate synapse turn-over [4]. On the other hand, chronic inflammatory activity of microglia exacerbates neuronal damage and prevents recovery [5]. However, once inflammation subsides, microglia can secrete anti-inflammatory cytokines such as IL-10 and TGF-β that can promote neuronal plasticity and repair [5, 6]. Thus, post-injury recovery is dependent on the interplay and balance of inflammatory responses and facilitation of neuronal synaptic plasticity, which is the key to developing effective therapeutics, but not well understood especially in the primate brain.

Recent studies from our group demonstrated that intravenous administration of extracellular vesicles (EVs) derived from bone marrow mesenchymal stromal cells (MSCs) facilitate recovery of motor function after cortical injury in the primary motor cortex (M1) [7–9]. EVs are nanovesicles containing various biomaterials including proteins, DNAs, RNAs and miRNAs, and are involved in cell-to-cell signaling [10, 11]. Intravenously administered EVs derived from MSCs in a rat model of traumatic brain injury enhanced spatial learning and sensorimotor functional recovery, increased vascular density, angiogenesis, and neurogenesis, promoted distal axon growth and reduced brain inflammation [9, 10]. In a series of studies from our group using our aged rhesus monkey model of cortical injury that involves induced damage in the hand representation of M1, we found that intravenous infusions of MSC-EVs 24 hours and again at 14 days post-injury enhanced recovery of fine motor function of the hand. Follow-up studies have shown that this EV-mediated recovery is due to promoting a shift from inflammatory to homeostatic microglial ramified morphologies, reducing injury-related excitotoxic neuronal hyperexcitability, and enhancing dendritic and synaptic plasticity in perilesional M1 and premotor cortex (PMC) [12–14].

The current study aims to build on these previous findings by assessing the interplay between the microglial-mediated responses and changes in neuronal synaptic plasticity. Specifically, we determined whether EV treatment alters the expression of synaptic markers for excitatory and inhibitory transmission, as well as structural and molecular markers of microglia-synapse interactions after cortical injury. In addition, we showed how microglia-synapse outcome measures are correlated with functional recovery. Specifically, we evaluated the co-expression of microglia and synapses with C1q, an integral protein that initiates the classical complement pathway cascade that may lead to synapse phagocytosis [15].We assessed tissue from perilesional M1 and intact dorsal PMC harvested from the same cohort of non-lesion monkeys and lesion monkeys with vehicle or EV treatment used in our previous studies [12–14]. Our results indicated that EV treatment enhanced anti-inflammatory microglial phagocytosis in perilesional M1 while dampened lesion-related synaptic loss by inhibiting excessive phagocytosis in the PMC, to therefore preserve synaptic activity and facilitate recovery of function.

## 2. MATERIAL AND METHODS

### 2.1 Animals and experimental design

A total of 13 Rhesus monkeys (*Macaca mulatta*), ranging from 16 to 26 years old (12 females, 1 male), acquired from either national primate centers (Emory or Tulane National Primate Research Centers) or private vendors (WorldWide Primates, Inc.), were used for this study. These monkeys were from the same cohort of non-lesion and lesion monkeys used in our previous studies [12–14]. All monkeys were prescreened to exclude animals with brain abnormality or history of neurological diseases, chronic diseases, diabetes, or malnutrition [16]. They were housed in the Animal Science Center of Boston University Medical Campus under a 12-hour light/dark cycle. All experimental procedures using animals were approved by the Boston University Institutional Animal Care and Use Committee (IACUC), and performed in accordance with the Guide for the Care and Use of Laboratory Animals from the NIH’s Offi ce of Laboratory Animal Welfare. Tissue from the lesion group were obtained from the cohort of monkeys used in our previous study [12]. These monkeys were trained on a fine motor task, the Hand Dexterity Task, for a total of four weeks and randomly assigned into the EV treated group (n = 5), vehicle group (n = 5), as described [12]. A surgical lesion was then made in the hand representation of the primary motor cortex (M1). Two weeks after the surgery, monkeys then began testing on the motor tasks for 12 weeks to assess the degree and nature of recovery. A separate cohort of aged-matched non-lesion monkeys (n = 3) which were part of a larger study on aging, was used as non-lesion control comparisons.

### 2.2 Surgical lesion of M1 hand representation and post-injury treatment

Surgical procedures to induce cortical injury were employed in the lesion cohort, as described [12, 14]. Briefly, each monkey was sedated with ketamine (10 mg/kg) and anesthetized with intravenous sodium pentobarbital (15–25 mg/kg). To limit the surgical lesion to the hand representation of M1 on the hemisphere controlling the dominant hand, the precentral gyrus was electrophysiologically mapped using a small silver ball surface stimulating electrode (with minimum current stimulus amplitude: 1–3 mA) [12, 14]. While the stimulating electrode was moved across the precentral gyrus, a trained observer recorded and graded the evoked muscle movements of the hand to create a cortical surface map of the hand area, as described [12]. To induce the lesion, a small glass suction pipette was inserted through an incision in the pia at the dorsal limit of the hand area, and then bluntly separated the penetrating arterioles from the underlying cortex.

At 24 hours and 14 days after the injury, monkeys were treated with either vehicle control or EV intravenously. The EVs were extracted from MSCs harvested from the bone marrow of a young adult monkey, as described [12, 14]. For bone marrow extraction, the monkey was sedated with ketamine (10 mg/kg) and anesthetized with intravenous sodium pentobarbital. Bone marrow extracted from the iliac crest was shipped to Henry Ford Health Systems, where the MSCs were isolated and cultured *in vitro* for EV collection, as described [9, 10, 12, 13]. EVs were then shipped back to Boston University for intravenous administration in monkeys (EVs were administered at 4 × 10^11^ particles/kg).

### 2.3 Perfusion and brain tissue Preparation

At 14 to 16 weeks after the injury, monkeys were sedated with ketamine (10 mg/kg), anesthetized with intravenous sodium pentobarbital (15–25 mg/kg) for perfusion and brain harvesting. Monkeys were perfused by using a two-stage Krebs-PFA perfusion method as described [14]. Ice-cold Krebs-Heinsleit buffer (6.4mM Na_2_HPO_4_, 1.4mM Na_2_PO_4_, 137mM NaCl, 2.7mM KCl, 5mM glucose, 0.3mM CaCl_2_, 1mM MgCl_2_, pH7.4) was used for perfusion and collecting fresh tissue biopsies. A tissue block just ventral to the lesion, containing mainly ventral premotor cortex (vPMC), was harvested from each monkey and then transferred to oxygenated (95% O_2_, 5% CO_2_) ice-cold Ringer’s solution (26mM NaHCO_3_, 124mM NaCl, 2mM KCl, 3mM KH_2_PO_4_, 10mM glucose, 1.3mM MgCl_2_, pH 7.4), as described [14]. This vPMC tissue block was sectioned into 300 μm coronal acute slices with a vibratome for *in vitro* whole-cell patch clamp recording and intracellular filling [14, 17].

The rest of the brain was fixed with 4L of 4% paraformaldehyde (30°C, pH7.4) and blocked *in situ* in the coronal plane [13]. The whole brain was then removed from the skull and cryoprotected in 0.1M phosphate buffer (PB) with 10% glycerol, and 2% DMSO, and then in buffer with 2% DMSO and 20% glycerol [13]. Brains were flash-frozen in −75°C isopentane and stored at −80°C before being cut on a microtome into interrupted series containing eight series of 30μm sections and one series of 60μm sections [13]. Sections were kept in the phosphate buffer with 15% glycerol and stored in −80°C for later processing.

### 2.4 Immunohistochemistry on serial sections through the lesion

To assess pre- and postsynaptic markers and structures after injury, we used immunohistochemical labeling on 30 or 60 μm coronal sections through the lesion (n = 1–2 sections per case) to quantify presynaptic vesicular glutamate (VGLUT1 and VGLUT2) and GABAergic (γ-aminobutyric acid) transporters (VGAT), as well as postsynaptic α-amino-3-hydroxy-5-methyl-4-isoxazolepropionic acid (AMPA) glutamate (GLUR2/3) and GABA (GABA_a_ *α*1, and GABA_b_ R2) receptor subunits. To assess the microglia activity, we immuno-labeled two microglia markers: Iba-1(ionized calcium binding adaptor molecule 1)—a pan microglial marker, and P2RY12 (purinergic receptor P2Y, G-protein coupled, 12)—a purigenic receptor associated with motility and homeostasis [18, 19]. We also immunolabeled C1q, an integral complement pathway protein that is an initiator of the classic complement pathway inflammatory cascade [15]. Previous studies in rodents suggest that upregulated C1q was associated with neuronal damage and microglial-mediated phagocytosis of synapses [20].

All the sections were incubated in 50mM Glycine for 1 hour and were washed with 0.01M PBS. Antigen retrieval was performed via incubation in 10mM citrate buffer (pH = 8.5) at 60–65°C for 20 minutes. Sections were then washed with 0.01M PBS, and incubated in the pre-block solution [5% bovine serum albumin (BSA), 5% normal donkey serum (NDS), 0.2% Triton X-100 in 0.01M PBS] for 1 hour at room temperature. Sections were incubated in primary antibodies in carrier solution (0.2% BSA, 1% NDS, 0.1% Triton X-100 in 0.1M PB) at 4°C for 72 hours. We used primary antibodies to goat VGLUT1 (1: 500, Synaptic Systems, Cat# 135307, RRID: AB_2619821), guinea pig VGLUT2 (1:1000, Synaptic Systems, Cat# 135404, RRID: AB_887884), rabbit GLUR2/3 (1:500, Millipore Cat# 07–598, RRID:AB_11213931), guinea pig VGAT (1:400, Synaptic Systems, Cat# 131004, RRID: AB_887873), rabbit GABA_a_ *α*1 (1:500, Abcam, AB33299, RRID: AB_732498), mouse GABA_b_ R2 (1:1000, LSBio, LS-C285897). For the microglia, we used a combination of rabbit P2RY12 (1:250, Novus Biologicals, NBP2–33870) and rabbit Iba-1(1:500, Wako, Cat# 019–19741, RRID: AB_839504) to enhance the staining of processes [21]. For C1q marker, we used mouse C1q (1:400, Abcam, AB71940, RRID: AB_10711046). Brain sections were washed with PBS and incubated for 24 hours at 4°C in donkey secondary IgG antibodies conjugated to fluorescence probes (1:200): AlexaFluor 488 donkey anti-guinea pig IgG (Jackson Immuno Research Labs, Cat# 706–545–148, RRID: AB_2340472), Alexa Fluor 546 donkey anti-goat IgG (ThermoFisher Scientific, Cat# A-11056, RRID: AB_2534103), Alexa Fluor 546 donkey anti-mouse IgG (ThermoFisher Scientific, Cat# A10036, RRID: AB_2534012) and biotinylated donkey anti-rabbit (Jackson Immuno Research Labs, Cat# 711–065–152, RRID: AB_2340593). During all primary and secondary incubations, sections were microwaved using the low-wattage PELCO Biowave (TED PELLA Inc, CA) 2 × 10 minutes (at 40°C, 150 watts) to aid in antibody penetration. In order to amplify the signal of Iba-1 + P2RY12, sections were incubated first in biotinylated donkey anti-rabbit secondary IgG followed by 24 hours in streptavidin 635 (1:200, Invitrogen). Autofluorescence was then reduced by incubating brain sections in 10mM cupric sulfate (50 mM ammonium acetate and 10 mM cupric sulfate in 10 ml deionized water) for 30 minutes. Before the final mounting and cover-slipping with the Prolong antifade mounting medium, the sections were washed with dH2O and PB to wash off excessive cupric sulfate.

### 2.5 Intracellular filling during *in-vitro* whole cell patch clamp recording and immuno-staining of microglia

To study microglia-spine interactions, we used pyramidal neurons intracellularly filled from whole-cell patch clamp recordings conducted for our previous study [14]. During Krebs perfusion, a fresh tissue block was harvested from vPMC and cut into 300-μm-thick coronal brain slices using a vibratome, which were then placed into room temperature oxygenated (95% O2, 5% CO2) Ringer’s solution. After 1 hour of equilibration period, individual brain slice was placed in submersion-type recording chambers (Harvard Apparatus), mounted on the stages of Nikon E600 infrared-differential interference contrast microscopes (Micro Video Instruments). Then, *in-vitro* whole cell patch clamp experiments and intracellular filling were performed on the layer 3 (L3) pyramidal neurons in the perilesional region at room temperature, as described [14, 17], to obtain electrophysiological data for our previous study [14]. Electrodes were fabricated on a horizontal Flaming and Brown micropipette puller (model P-87, Sutter Instruments) [17]. Potassium methanesulfonate-based solution (concentrations in mM: 122 KCH3SO3, 2 MgCl2, 5 EGTA, 10 Na- HEPES, pH 7.4; Sigma-Aldrich) with 1% biocytin was used as internal solution in electrodes (resistances of 3– 6 MΩ) to fill the pyramidal neurons. After recording, slices were fixed in 4% PFA for 2 days. In order to visualize the cells filled with biocytin, slices were incubated in 1% Triton-X in 0.1M PB for 2 hours at room temperature, and in streptavidin-Alexa 488 for 2 days (1:500 in 0.1 M PBS, Invitrogen). After recording, slices were fixed in 4% PFA for immunolabeling experiments.

To assess microglia-spine appositions, slices with filled cells were then processed for immunolabeling of microglia. Slices were first incubated in 50mM Glycine for 1 hour, followed by antigen retrieval with a 20-min incubation in 10mM citrate buffer (pH = 8.5) at 60–65°C. To label microglia, a combination of primary antibodies to Iba-1 (1:500, Wako, Cat# 019–19741, RRID: AB_839504) and P2RY12 (rabbit, 1:250, Novus Biologicals, NBP2–33870) was diluted in a carrier solution (0.2% BSA, 1% NDS, 0.1% Triton X-100 in 0.1M PB). Slices were incubated for 7 days at 4°C in primary antibodies, with 2 × 10 min low wattage microwave sessions (150 W, 40°C in Ted Pella biowave) on days 1, 2, 4 and 5, to assist with antibody penetration. Slices were then incubated in anti-rabbit IgG secondary antibody conjugated to Alexa 546 for 4 days at 4°C with 2 × 10 min microwave sessions (150 W, 40°C) were performed on days 1 and 3.

### 2.6 Confocal imaging and quantification of synaptic markers

**Figure 1:**
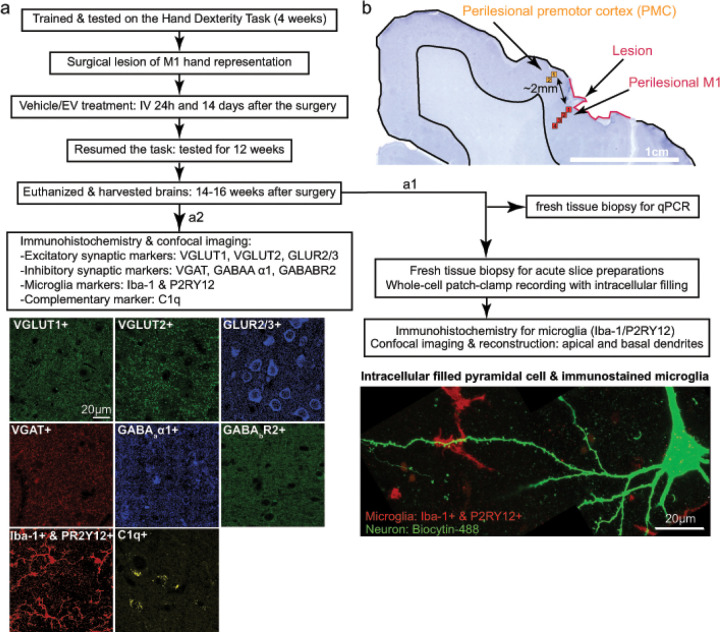
Experimental design and representative Images of immunolabeled markers, lesion, and sampling location. **a** Experimental workflow: monkeys were trained and tested on a fine motor task – the Hand Dexterity Task, before and after the surgical lesion of M1 as described in Moore et al., 2019. Then, the monkeys were randomly assigned with either vehicle or EV treatment, which were infused IV at 24 hours and 14 days post-injury. The brains were harvested 14 to 16 weeks after the surgery. (*a1*) During Krebs buffer perfusion, 1–2cm fresh tissue block was harvested from the ventral perilesional motor/premotor cortex: the caudal 1/4 was processed for qPCR and the rostral part was cut into 300 μm acute slices for whole cell patch clamp recording and intracellular filling of layer 3 pyramidal cells. *(a2)* The remainder of the brain containing the lesion and rostral and dorsal perilesional cortex was fixed with paraformaldehyde then cut into serial coronal sections and processed with immunohistochemical labeling. The synaptic markers that were immuno-labeled included: VGLUT1 &2, GLUR2/3, VGAT, GABA_a_ *α*1, and GABA_b_ R2. Microglia markers were combining labeled Iba-1 and P2RY12. C1q was immuno-labeled to indicate complement activation. Pyramidal neurons were intracellularly filled with internal solution with 1% biocytin. **b** Sample image of surgical lesion and sampling location: perilesional primary motor (M1) and premotor cortices (PMC)

All 30 or 60 μm sections were imaged by a Leica TCS SPE laser scanning confocal microscope with 3 laser lines: 488 nm, 546 nm, and 647 nm (Leica Microsystems). Sections were imaged with a 40× 1.3 N.A. oil objective lens at a resolution of 0.134*0.134*0.5μm. We imaged four fields, spaced 400μm apart, directly underlying the damaged pial surface (from ~ 200 μm to ~ 1200 μm distal to the damaged pial surface) in M1 gray matter, and two fields in PMC gray matter layer 2/3 with intact pial surface ~ 2mm distal to the lesion in each brain section (Fig. 1b). Each confocal image was deconvolved using AutoQuant (Media Cybernetics) and converted to 8-bit images for further analysis.

To quantify the synaptic markers, we analyzed the optical density (percent area labeled) and size of immunolabeled pre- and postsynaptic markers using particle analysis function in FIJI/ImageJ (https://imagej.net/Fiji;1997–2016;RRID:SCR_002285) [19]. The signal threshold for analysis was set with the Renyi method of FIJI in the first field (layer ½) and applied to all the other fields of the same section. The physical contacts, or colocalization, between synaptic markers (VGLUT1 & 2, VGAT) and microglial markers (P2RY12 & Iba-1) or complementary marker C1q were analyzed by using the co-localization plugin of FIJI/ImageJ. The percent area of co-locolization was first obtained and then a colocalization coeffi cient was calculated based on Mander’s method (the percent area colocalized/percent area of marker 1 or maker 2). The average measures of synaptic puncta optical density and microglia-synapse colocalization were calculated for each animal and compared between groups.

### 2.7 Quantification of microglia-neuron interactions

We assessed the interaction between filled L3 pyramidal neurons and immuno-labeled microglia in the perilesional ventral premotor cortex. Dual channel imaging was conducted using a Leica TCS SPE laser scanning confocal microscope with 488 nm and 546 nm laser under 63x/1.4 N.A. oil objective lens at a resolution of 0.04*0.04*0.3μm. One apical dendrite and one basal dendrite of each filled cell were followed and scanned from base to tip. Confocal scanned images were montaged, and the dendritic segments were traced and reconstructed in Neurolucida 360 (RRID: SCR_016788; MBF Bioscience). The appositions of microglia (P2RY12/Iba-1+) on dendrites and dendritic spines were counted and categorized as contacts and neighboring. Contacts required overlap of saturated signal from the two channels, while neighboring was identified when the signal of two channels were adjacent and the distance was ~ 0.3μm to ~ 1μm. The traced dendrites with markers of microglial apposition were analyzed and exported using Neuroexplorer (v11.01, Microbrightfield). The density of appositions (# of appositions/total length of dendrite imaged) was calculated for each dendrite.

### 2.8 Microglia classification and reconstruction

We used the NeuroLucida 360 software (RRID: SCR_016788; MBF Bioscience) to classify and reconstruct the microglia. We examined each field through the entire Z-stack and counted the microglia by marking them based on their morphological phenotypes [13, 22]. Specifically, microglia were classified into three categories based on morphology: 1) ramified microglia were classified based on the appearance of a round soma, and thin multipolar primary process. 2) Hypertrophic I microglia were classified characterized by slightly enlarged (about 1.5x larger than ramified) and ovoid somata with slightly thickened processes (about 2x thicker than ramified). 3) The amoeboid/hypertrophic II had either large somata (> 2x more than ramified) with very thick and short process, or with highly elongated somata with thick processes almost as thick as the somata diameter. The microglia identified by Iba-1 staining and classified by morphology, were then classified based on C1q expression, thus allowing us to quantify six categories of microglial phenotypes as follows: ramified (Rami) C1q negative cells (R-), ramified cells with C1q colocalized in the soma or processes (R+), hypertrophic (Hyper) C1q negative cells (H-), hypertrophic cells with C1q colocalized in the soma or processes (H+), amoeboid (Ame) C1q negative cells (A-) and amoeboid cells with C1q colocalized in the soma (As+).

We then reconstructed the soma and primary process of a subset of the classified microglia. Somata were reconstructed in NeuroLucida 360 (Microbrightfield, Inc.), using the 3D environment and soma auto- detection features. The soma detector sensitivity was maintained between 70–90, the interactive search region ranged between 20–30 μm, and the size constraint was between 2–5 μm. For the somas that were not able to be auto-detected using the above measurements, we manually contoured them by using the Cell Body trace feature in the software and focusing through the Z-stack. In addition, all the primary processes of the microglia were manually contoured using the Dendrite feature since we wanted their thickness to be accurately determined. Once all the microglia were classified and reconstructed, we exported the data for microglia using the NeuroLucida Explorer (v11.01, Microbrightfield).

### 2.9 RNA isolation and qPCR

Ventral perilesional brain tissue containing the caudal PMC/M1 area of each animal was dissected at euthanasia (14 weeks post-injury), flash-frozen using dry ice, then stored at −80◦C until RNA isolation as described in our previous work [13]. Briefly, tissue samples were thawed on dry ice and dissected into 100 mg pieces for each animal before mechanical homogenization using an RNAse free scalpel. Then, tissue samples were chemically triturated using the TRIzol method as follows (ThermoFisher, Waltham, MA); briefly, tissue was placed in TRIzol and passed through an 18-gauge needle to further homogenize tissue. An organic extraction was then performed using chloroform and ethanol, according to the manufacturer’s protocol (ThermoFisher, Waltham, MA). The extracted RNA was then air-dried and resuspended in 40 μL of PCR-grade water. RNA purity was checked using UV absorbance ratio at A260/280 with a NanoDrop spectrophotometer (ThermoFisher, Waltham, MA).

After extraction, RNA was converted to cDNA using a High-Capacity RNA-to-cDNA kit (ThermoFisher, Waltham, MA), according to the manufacturer’s protocol, then normalized to 2 μg for each sample. Gene expression analysis was performed in triplicate using quantitative polymerase chain reaction (qPCR) with either Taqman Gene Expression Assay or PowerUp Sybr Green Master Mix (ThermoFisher, Waltham, MA). Relative gene expression levels of *GRIA1* (AMPA GLUR1), *GRIA2* (AMPA GLUR2), *GRIN1* (NMDA NR1), *GRIN2B* (NMDA NR2B), *GABRA1* (GABA_a_ *α*1), *GABRA2* (GABA_a_ *α*2), *GABRA5* (GABA_a_ *α*5), *GABRD* (GABA_a_ ∂), *GABBR2* (GABA_b_ R2), *C1qA* (C1q) and C3 (C3) were calculated using the ΔΔCt method with *GAPDH* as reference gene for normalization.

### 2.10 Statistical analysis

All data were expressed as box-and-whisker plots and linear plots with means. Statistical analyses were processed in MATLAB (R2020a, MathWorks, Natick, MA) to calculate the average of each field and each animal. The outcome measures of each synaptic marker and colocalization were compared between groups using one-way ANOVA, with post hoc Fisher’s LSD in MATLAB. For pairwise comparison of groups with lesion, Student’s *t* tests were performed for measures of synaptic markers, colocalization, microglia-spine contacts, and qPCR results. Linear correlations between variables were determined using linear regression analyses in MATLAB. Nonmetric multidimensional scaling (NMDS) was performed to analyze similarities among three groups (non-lesion control, veh, EV) based on 9 gene expression outcome variables from qPCR analyses (*GRIA1*, *GRIA2*, *GRIN1*, *GRIN2B*, *GABRA1*, *GABRA2*, *GABRA5*, *GABRD*, *GABBR2*), and 21 synaptic and microglia outcome measures (%area VGLUT1, VGLUT2, VGAT, GLUR2/3, GABA_a_ *α*1, GABA_b_ R2; % of VGLUT1, VGLUT2 or VGAT with Iba-1; % of Iba-1 with VGLUT1, VGLUT2 or VGAT; % area C1q; % of VGLUT2 with C1q; % C1q with VGLUT2; cell densities of Ramified, Hypertrophic, Amoeboid C1q + and C1q- microglia), as described [14]. Z-scores were obtained for each variable, and pair-wise comparisons were used to calculate a distance matrix based on squared Euclidean distances. The multidimensional distance matrix was then reduced to two dimensions via NMDS, and the resulting values of each case were plotted, with the distances between data points representing the relative similarities based on the set ofvariables. For the results of microglia reconstruction, 3-way ANOVA with post hoc Fisher’s LSD was performed in MATLAB for soma volume and aspect ratio, using three factors: group, phenotype/morphology, and C1q+/−.

## 3. RESULTS

### 3.1 Lesion-related reduction of VGLUT2 but not VGLUT1 density in perilesional M1 and PMC

**Figure 2:**
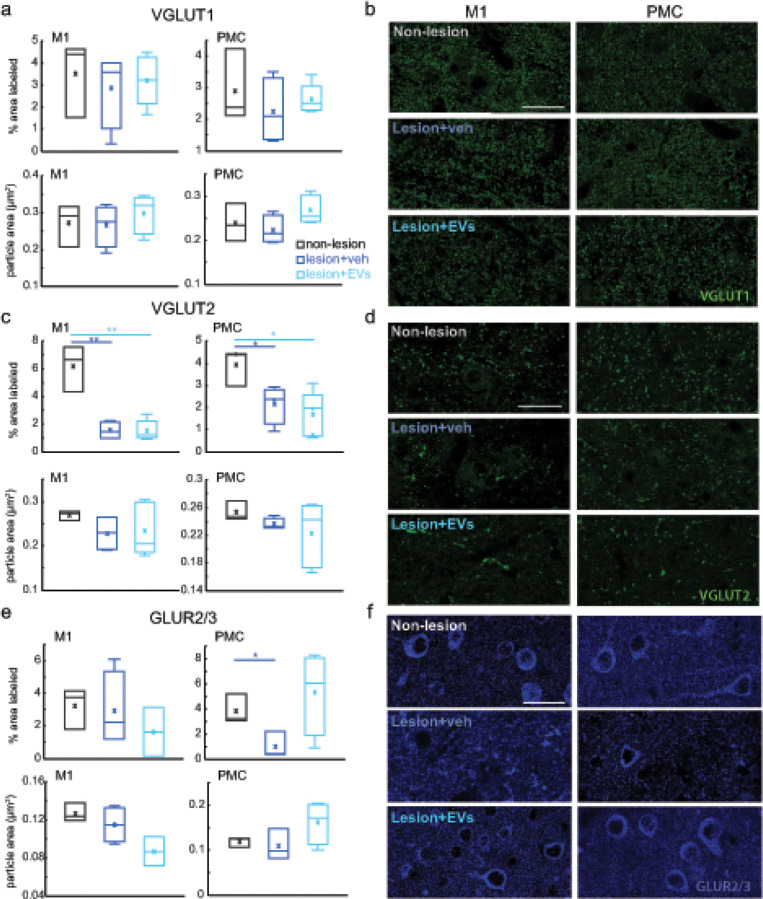
The expression of excitatory synaptic markers in perilesional M1 and PMC. **a** The density (% area labeled) and average particle size (area in μm^2^) of VGLUT1+ puncta in perilesional M1 and in PMC. Non-lesion control: n=3. Veh group: n=4. EV group: n=5. **b** Representative maximum-projection confocal images of VGLUT1 immuno-labeling in M1 and PMC. Scale bar: 20μm. **c** The density and average size of VGLUT2+ puncta in perilesional M1 and in PMC. The density of VGLUT2+ puncta was significantly lower in groups with lesions compared with non-lesion controls (con) in both perilesional M1 (Fisher’s LSD *post hoc*, con. vs veh: p=0.004; con vs EV: p=0.001) and in PMC (Fisher’s LSD *post hoc*, con. vs veh: p=0.04; con vs EV: p=0.02). Non-lesion control: n=3. Veh group: n=4. EV group: n=5. **d** Representative maximum-projection confocal images of VGLUT2 immuno-labeling in M1 and PMC. Scale bar: 20μm. **e** The density (% area label) and average size (μm^2^) of GluR2/3+ puncta in perilesional M1 and in PMC. The density of GLUR2/3+ puncta in PMC was significantly lower in the vehicle group (Fisher’s LSD *post hoc*, con. vs veh, p=0.036) but not in the EV group (Fisher’s LSD *post hoc*, con. vs EV, p=0.49) as compared with non-lesion controls. Non-lesion control: n=3. Veh group: n=4. EV group: n=4. **f** Representative maximum-projection confocal images of GLUR2/3 immuno-labeling in M1 and PMC. Scale bar: 20μm

To assess the extent of synapse loss and remodeling with lesion and EV treatment, we determined the optical density (% area labeled) and size of immunolabeled presynaptic and postsynaptic structures in perilesional M1 gray matter and perilesional dorsal PMC (Fig. 1b). Using one-way ANOVA, we assessed the effect of experimental group (non-lesion, lesion + veh and lesion + EV) on the expression of presynaptic axon terminals labeled with VGLUT1 and VGLUT2, which represent two distinct sets of inputs to the cortex. VGLUT1 is known to label mostly terminals from cortico-cortical pathways, while VGLUT2 labels axon terminals from subcortical afferents, mainly the thalamus [23]. We found a significant effect of lesion on the density of VGLUT2 + axon terminals in both perilesional M1 and dorsal PMC (Fig. 2c–d): both EV and veh lesion groups showed significantly lower density (% area labeled) of VGLUT2 + puncta compared to the non-lesioned controls, indicating a lesion-related loss of VGLUT2 in M1 and PMC (Fig. 2c: Fisher’s LSD *post hoc*, M1: p < 0.01; PMC: p < 0.05). In contrast to differences in VGLUT2, VGLUT1 density was not significantly impacted by the lesion (Fig. 2a–b). No significant treatment effect was found for the density of either VGLUT1 + or VGLUT2 + puncta.

### 3.2 EV treatment mitigated the lesion-related reduction of postsynaptic GLUR2/3 in PMC

In order to assess the effects of lesion and EV treatment on excitatory postsynaptic structures, we analyzed the density of GLUR2/3 AMPA glutamatergic receptor subunits in perilesional M1 gray matter and PMC. The AMPA receptor is the ionotropic glutamate receptor responsible for fast excitatory synaptic transmission [24]. In PMC, GLUR2/3 subunit density was significantly reduced in the vehicle treated group compared with non-lesion controls (Fig. 2e: Fisher’s LSD *post hoc*, p < 0.05), indicative of a lesion-related decrease in efficacy of AMPA synaptic transmission (Fig. 2e–f). In contrast, the EV treated group did not differ from the non-lesion group in GLUR2/3 subunit density (Fig. 2e–f). The lesion resulted in a reduction of postsynaptic GLUR2/3 in perilesional cortices, which was ameliorated by EV treatment.

### 3.3 Lesion-related dysregulation of GABAergic postsynaptic receptor subunit expression in perilesional M1 and PMC

**Figure 3:**
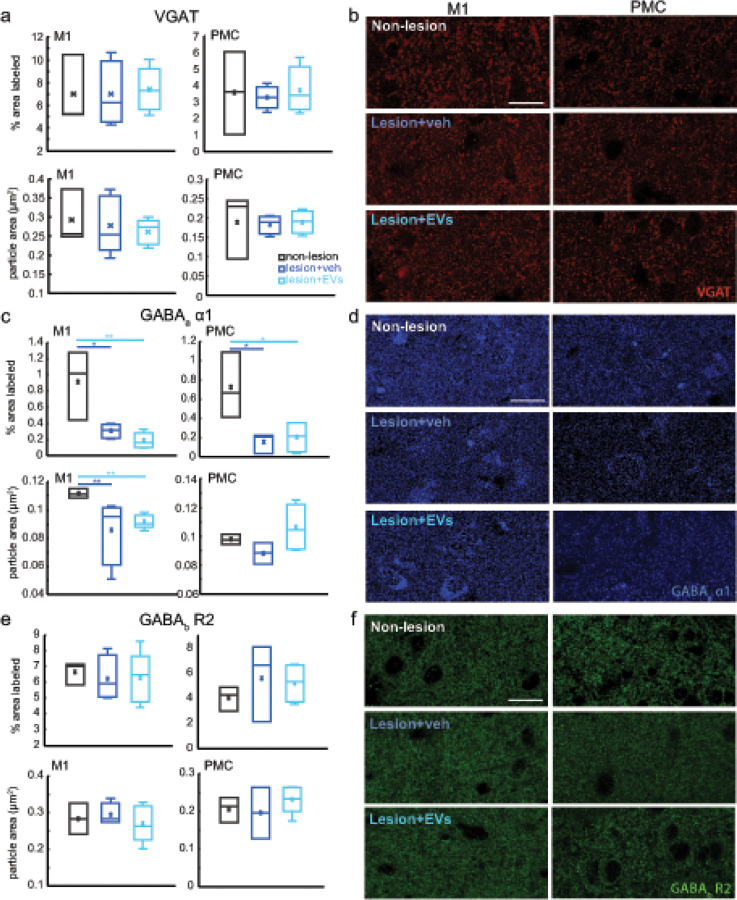
The expression of inhibitory synaptic markers in perilesional M1 and PMC. **a** The density and average size of VGAT in perilesional M1 and in PMC. No significant result was found. Non-lesion control: n=3. Veh group: n=5. EV group: n=5. **b** Representative maximum-projection confocal images of VGAT immuno-label in M1 and PMC. Scale bar: 20μm. **c**. The density and average size of GABA_a_ *α*1 in perilesional M1 and in PMC. The density of GABA_a_ *α*1 subunit in perilesional M1 was significantly lower in both veh (Fisher’s LSD *post hoc*, con. vs. Veh, p=0.038) and EV group (Fisher’s LSD *post hoc*, con. vs. EV, p=0.009) as compared with non-lesion controls. The density of GABA_a_ *α*1 in PMC was lower in veh (Fisher’s LSD *post hoc*, con. vs. veh, p=0.05) and EV group (Fisher’s LSD *post hoc*, con. vs. EV, p=0.02). The size of GABA_a_ *α*1 in M1 was significantly smaller in the EV group (Fisher’s LSD *post hoc,* con. vs. EV, p<0.001). Non-lesion control: n=3. Veh group: n=4. EV group: n=5. **d** Representative maximum-projection confocal images of GABA_a_ *α*1 receptor subunits immuno-label in M1 and PMC. Scale bar: 20μm. **e**. The density and particle average size of GABA_b_ R2 subunit in perilesional M1 and in PMC. Non-lesion control: n=3. Veh group: n=4. EV group: n=5. **f** Representative maximum-projection confocal images of and GABA_b_ R2 subunits immuno-label in M1 and PMC. Scale bar: 20μm

We assessed the effects of lesion and EV treatment on inhibitory synapses by analyzing the density of immunolabeled presynaptic VGAT and postsynaptic GABA_a_ *α*1 and GABA_b_ receptor 2 subunits. In contrast to lesion effects on excitatory presynaptic terminals, no significant between-group difference was found for presynaptic VGAT in perilesional M1 or PMC gray matter (Fig. 3a–b). However, significant lesion effects were found on the expression of distinct postsynaptic GABA receptor subunits. Post-synaptically located GABA_a_ *α*1 subunits are associated with ionotropic receptors responsible for fast inhibitory synaptic transmission, whereas GABA_b_ R2 subunits are associated with metabotropic receptors that mediate slow or tonic inhibition [25–27]. There was a significant lesion-related reduction of density of GABA_a_ *α*1 + puncta in both perilesional M1 and PMC (Fig. 3c–d. M1: Fisher’s LSD *post hoc*, con. vs. veh, p < 0.05; con. vs. EV, p < 0.01; PMC: Fisher’s LSD *post hoc*, con. vs. veh, p = 0.05; con. vs. EV, p < 0.05). In perilesional M1, both groups with lesion had smaller average size of GABA_a_ *α*1 + puncta (Fig. 3c: two-way ANOVA, main effect, p < 0.001). However, in PMC, the EV treated group showed a trend of larger GABA_a_ *α*1 puncta than vehicle group (Fig. 3c, Fisher’s LSD *post hoc*, veh vs. EV, p = 0.1).

### 3.4 EV treatment normalized gene expression of glutamate and GABA receptor subunits in PMC

**Figure 4:**
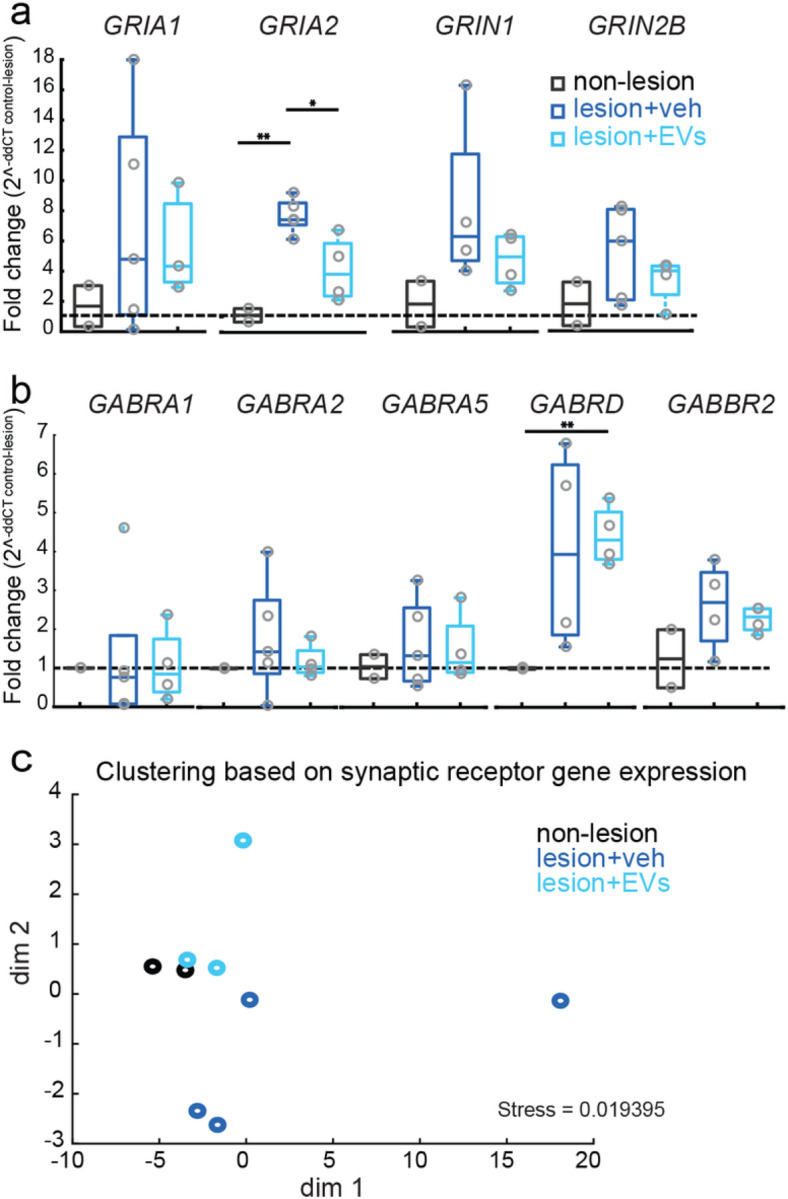
Glutamate and GABA receptors subunit mRNA expression in perilesional cortex. **a** Fold changes of glutamate receptor subunit gene expression. The gene expression of *GRIA2* (GluR2) was significantly higher in the veh group, as compared with non-lesion controls (*t*-test, p < 0.001) and the EV group (*t*-test, p=0.01). **b** Fold changes of GABA receptor subunit gene expression. The gene expression of *GABRD* (GABA_a_ ∂) was significantly higher in the EV group as compared with non-lesion controls *(t*-test, p=0.004). Gene names: *GRIA1* (AMPA GluR1), *GRIA2* (AMPA GLuR2), *GRIN1* (NMDA NR1), *GRIN2B* (NMDA NR2B), *GABRA1* (GABAa *α*1), *GABRA2* (GABAa *α*2), *GABRA5* (GABAa *α*5), *GABRD* (GABAa ∂), *GABBR2* (GABAb R2). **c** MDS plot showing clustering of cases based on mRNA expression profiles of Glu and GABA receptor subunits. The proximity of points indicates the relative similarity-based pair-wise correlation of these multiple mRNA expression variables

We also assessed whether the lesion or EV treatment impacted the transcription of glutamate and GABA receptors subunits by using qPCR. T-tests were used to compare between groups. Our results showed a lesion-related increase in *GRIA2* (AMPA GLUR2) mRNA expression, which was reduced by EV treatment (Fig. 4a, con vs. veh: p < 0.01; veh vs. EV: p < 0.05). A similar pattern of expression was found for *GRIN1* (NMDA NR1), although the differences among groups did not reach statistical significance (Fig. 6a, con vs. veh: p = 0.06, con vs. EV: p = 0.14).

Interestingly, our results showed a lesion-related increase in gene expression of *GABBR2* (GABA_b_ R2) Interestingly, our results showed a lesion-related increase in gene expression of GABBR2 (GABAb R2) and *GABRD* (GABA_a_ ∂), which transcribe GABA receptor subunits that mediate tonic inhibitory currents (Fig. 4b, p < 0.01). The NMDS plot (Fig. 4c) showed that veh and EV treated monkeys formed distinct clusters. Further the EV-treated cluster overlapped with non-lesion control, indicating that EV treatment shifted gene expression of GLURs and GABARs towards the non-lesion control expression pattern. In contrast, vehicle-treated lesion monkeys were more dissimilar (clustered farther in the NMDS plot) to non-lesion control based on GLURs and GABARs gene expression. These data suggest that EV treatment results in a “normalization” of lesion-related changes in gene expression of AMPA GLURs and GABARs.

### 3.5 Lesion-related increase in microglia interaction with synaptic elements

**Figure 5:**
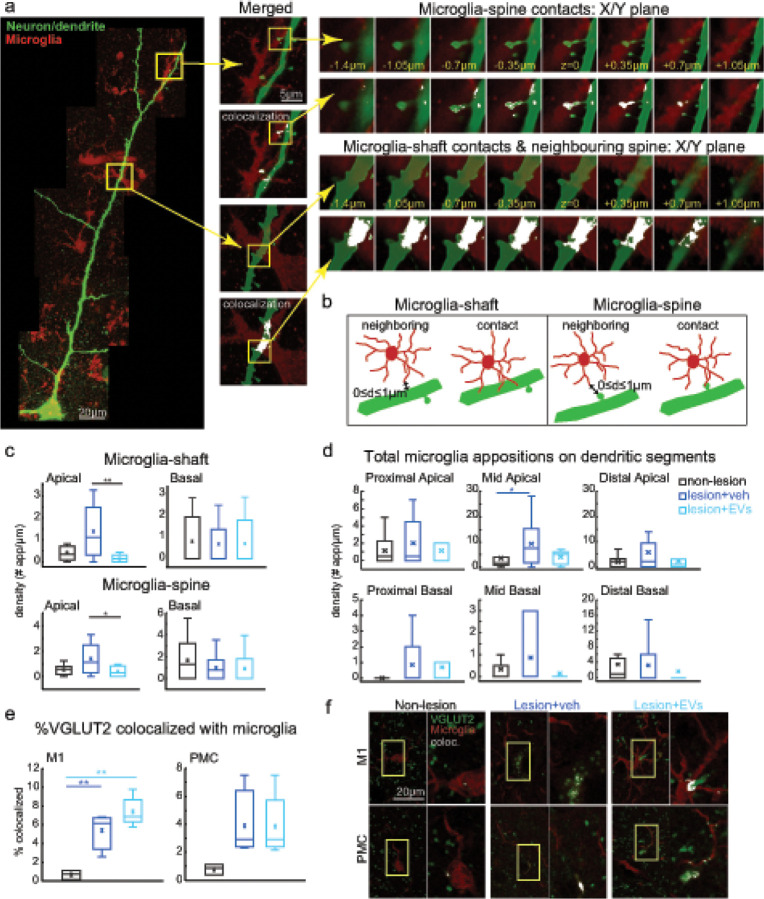
Microglia apposition on synaptic structures. **a** Representative images of microglia interactions with dendritic spines/shaft at different z-levels of stack. Neuronal dendrites and spines were filled with biocytin and stained with Streptavidin-Alexa 488 (green) and microglia were visualized with Iba-1 & P2RY12 + (red) immuno-labeling. White pixels indicate an overlap between two channels. Scale bar: 20μm and 5μm. **b** Schematic diagram of criteria for determining microglia appositions on dendritic shafts or spines, classified as either contact (touching) or neighboring (within 1 microns). **c**. The density of microglia appositions (contacting & neighboring) on dendritic shaft and spine. The vehicle group had higher densities of microglial-apical shaft (Fisher’s LSD *post hoc*, veh vs. EV, p=0.009) and microglial-apical spine (p=0.038) appositions compared to EV, and a trend for greater total appositions in the vehicle than in the control was found (con. vs. veh., p=0.058). **d** Total microglial appositions (contacts & neighboring on spines and shafts) on different segments of apical/basal dendrites in vPMC. Mid-apical dendrites had higher density of microglial contacts only in veh compared to control (Fisher’s LSD *post hoc,* con. vs veh, p=0.03; con. vs EV, p=0.84). Non-lesion control: n=10 from 3 monkeys. Veh group: n=10 from 3 monkeys. EV group: n=7 from 2 monkeys. **e** The fraction of VGLUT2 colocalized with microglia was higher in both groups with lesions in M1 (Fisher’s LSD *post hoc*, con. vs. veh, p=0.009; con. vs. EV, p<0.001). Non-lesion control: n=3. Veh group: n=4. EV group: n=5. **f** Representative images of dual channel labeling (left panel) of microglia (red) and VGLUT2 (green), with right panel showing higher resolution of colocalized VGLUT2-microglia label masked in white

Based on our finding of lesion-related decreases in excitatory VGLUT2 + presynaptic axon terminals (Fig. 2c–d) and postsynaptic GLUR2/3 expression (Fig. 2e–f), we then investigated the role of microglia in the processes of synaptic remodeling with lesion and EV treatment. Microglia has been suggested to closely appose synaptic elements in order to either phagocytose damage or release neurotrophic factors [4]. First, we assessed microglia interactions on the post-synaptic structures (dendritic shaft and spines), by quantifying close appositions (including contacts and neighboring) between Iba-1/P2RY12 + microglial processes and dendrites of intracellularly filled L3 pyramidal neurons in vPMC. A microglia-dendrite apposition was defined as a “contact” when microglial process directly overlaps with the dendrite spine or shaft, or as a close “proximity/neighboring” interaction when the microglial process was within 1 micron from the labeled neuronal dendritic spine or shaft (Fig. 5a–b). For the total microglia-dendrite appositions, which includes both direct contacts and neighboring interaction between microglia and dendrites (shafts and spines), the proportion of microglial appositions with spines and shafts were about equal (microglia-spine: ~40–60%). However, both microglia-spine and microglia-shaft interactions exhibited significant between-group differences, specifically on apical but not basal dendrites (Fig. 5c). Apical dendrites from neurons in vehicle group exhibited significantly higher density [#of appositions/100 μm dendrite length] of microglia-shaft interaction than those from EV group (Fig. 5c, Fisher’s LSD *post hoc*, veh vs. EV, p < 0.01), as well as a higher density of microglia-spine interaction than those from EV group, with a similar increasing trend as compared to the controls (Fig. 5c, Fisher’s LSD *post hoc*, con. vs. veh, p = 0.06; veh vs. EV, p < 0.05). Further, when looking at specific compartments, we found that the mid-apical dendritic segments had significantly higher density of microglia-dendrite appositions in pyramidal neurons of vehicle but not EV group, compared with non-lesion control group (Fig. 5d, Fisher’s LSD *post hoc*, con. vs veh, p < 0.05; con. vs EV, p = 0.84).

We then assessed the overlap of markers of microglia, Iba-1 and presynaptic VGLUT2 + axon terminals. The fraction of VGLUT2 colocalized with Iba-1 was greater in both groups with lesions in perilesional M1 (Fig. 5e–f, Fisher’s LSD *post hoc*, p < 0.01) compared to non-lesion controls.

These results suggest that the lesion was associated with increased microglial appositions on presynaptic VGLUT2 + terminals and postsynaptic structures (dendrites and spines) in perilesional motor cortices, and EV treatment reduced the lesion-related increase in microglial contacts on apical dendrites.

### 3.7 Lesion-related increase in C1q complement receptor expression on VGLUT2 + axon terminals and microglia

**Figure 6:**
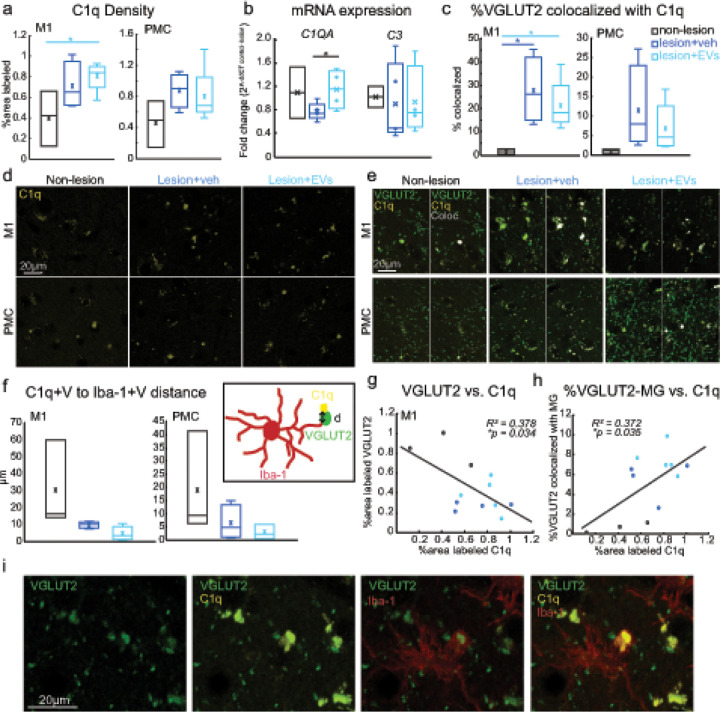
C1q co-expression on VGLUT2+ axon terminals and Iba1 microglia. **a** The density (% area label) of C1q+ puncta in perilesional M1 and PMC (Fisher’s LSD *post hoc*, M1: con. vs EV p=0.028; Non-lesion control: n=5. Veh group: n=5. EV group: n=4). **b** Fold changes of *C1QA* and *C3* gene expression in perilesional M1 (*t*-test, veh. vs. EV, p=0.027). **c** The density (% area label) of VGLUT2+ puncta colocalized with C1q+ puncta (Fisher’s LSD *post hoc*, con. vs. veh, p=0.02; con. vs. EV, p=0.02; Non-lesion control: n=3. Veh group: n=4. EV group: n=5). **d** Representative maximum-projection confocal images of C1q+ puncta immuno-labeling in M1 and PMC. Scale bar: 20μm. **e** Representative maximum-projection confocal images of 4 optical slices showing dual channel labeling (left panel) of C1q (yellow) and VGLUT2 (green), with colocalized VGLUT2–C1q points masked in white (right panel). Scale bar: 20μm. **f** The distance between the C1q-VGLUT2 colocalized points and microglia (Iba1)-VGLUT2 colocalized points. Inset shows schematic diagram of how C1q-VGLUT2-Iba1 distance was determined. Non-lesion control: n=3. Veh group: n=4. EV group: n=5. **g** Linear regression showing increasing C1q expression correlated with decreasing expression of VGLUT2+ (*R*^*2*^ = 0.378, p=0.034) in M1. **h** Linear regression greater C1q expression correlated with greater VGLUT2-Microglia colocalization (*R*^*2*^ = 0.372, p=0.035). **i** Representative maximum-projection confocal images of 9 optical slices showing single/dual/triple channel labeling (left to right) of colocalized VGLUT2-Iba1–C1q

The finding of lesion-related reduction of pre- (VGLUT2) and postsynaptic (GLUR2/3) markers coupled with a lesion-related increase in microglia appositions with these synaptic elements suggested a role of microglia in synapse phagocytosis and/or pruning. C1q, as a complement protein, contributes to synapse elimination by initiating the classical complement cascade [28]. In particular, C1q tags apoptotic cells or cellular debris, including damaged synapses, and triggers downstream signaling for phagocytic elimination by macrophages or microglia [28, 29]. We immuno-labeled C1q to further assess whether the lesion-related increase in these presumed “microglia-synapse interaction” is associated with synapse phagocytosis. In perilesional M1, the density of C1q + puncta was significantly higher in the EV group than in the non-lesion control group (Fig. 6a, d, M1: Fisher’s LSD *post hoc*, con. vs. EV: p < 0.05) while no significant between-group difference was found in PMC. Similarly, *C1QA* transcript levels in the ventral perilesional M1 was significantly greater in EV compared to vehicle group (Fig. 6b, qPCR fold change, t-test, veh. vs. EV, p < 0.05). Interestingly, no significant between-group differences were found with regards to mRNA transcript levels of *C3*, a downstream target of C1q. Overall, these data suggest an upregulation of C1q activity near the lesion, which was enhanced by EV treatment. This C1q upregulation was not associated with a downstream upregulation of C3 receptor pathway, suggesting involvement of a different complement pathway cascade. Further, it remained unclear if this EV-mediated C1q upregulation reflects increased C1q tagging of damaged synapses or increased C1q expression within microglia.

To estimate the synapses tagged with C1q, we assessed the colocalization between VGLUT2 + and C1q + puncta (Fig. 6c, e). We found a significant lesion-related increase in the fraction of VGLUT2 + puncta colocalized with C1q + in M1 in both treatment groups (Fig. 6c, e, M1: Fisher’s LSD *post hoc*, con. vs. veh, p < 0.05; con. vs. EV, p < 0.05). In M1, about 20–40% of all VGLUT2 + puncta were expressing C1q in the lesion groups, compared to the non-lesion group where only virtually no VGLUT2 + puncta expressed C1q. This lesion-related increase in the fraction of VGLUT2 + tagged with C1q was not significant in PMC (Fig. 6c, e, PMC: Fisher’s LSD *post hoc*, con. vs. veh, p = 0.17; con. vs. EV, p = 0.15), suggesting that C1q tagging of presynaptic VGLUT2 + terminals were prevalent within the M1 area most proximal to the lesion, and was diminished in areas more distal to the lesion. We then assessed whether these C1q + VGLUT2 + puncta were associated with and were within the vicinity of microglia-VGLUT2 contacts (Fig. 6f, i). Thus, we estimated the distance between C1q + VGLUT2 + puncta and microglia Iba-1 + VGLUT2 (C1q + V-Iba-1 + V distance) contacts (Fig. 6f). We found in M1 but not PMC, a trend of shorter C1q + V-Iba-1 + V distance in EV group as compared to the control and vehicle groups (Fig. 6f, Fisher’s LSD *post hoc*, M1: con. vs. EV, p = 0.06; veh. vs. EV, p = 0.06). These results suggested that within perilesional M1, microglial contacts on VGLUT2 + synapses were associated with the C1q tagging on these terminals, which implied an EV-related upregulation of microglial phagocytosis of VGLUT2 + synapses within the area nearest to the lesion.

### 3.8 Relationship of lesion related VGLUT2 loss to microglia C1q expression

Given that we found decreased synaptic marker expression and corresponding increased VGLUT2–C1q colocalization and microglia interaction associated with lesion, we therefore used linear regression to assess relationships between C1q and VGLUT2 densities, and VGLUT2, C1q and microglia (MG) co-localization (Fig. 6g–i). In M1, the decreased density of VGLUT2 + puncta was associated with increased density of C1q + puncta (Fig. 6g, *R*^*2*^ = 0.378, *p* < 0.05), and increased fraction of VGLUT2 tagged by C1q (not shown, *R*^*2*^ = 0.536, *p* < 0.01). Further, increased microglia-VGLUT2 co-localization in M1 was associated with increasing density of C1q + puncta (Fig. 6h, *R*^*2*^ = 0.372, *p* < 0.05), shorter distance between C1q tag and microglia-VGLUT2 contacts (not shown, *R*^*2*^ = 0.474, *p* < 0.05), and more C1q-Iba-1 colocalization (not shown, *R*^*2*^ = 0.725, *p* < 0.001). These data together indicated that the higher expression of C1q and increased C1q tagging of VGLUT2 + in perilesional M1 was associated with loss of VGLUT2 + and increased VGLUT2–C1q-microglia interaction after lesion, which implicated an increase in microglial synaptic phagocytosis.

### 3.9 EV treatment increased C1q expression in hypertrophic microglia

**Figure 7:**
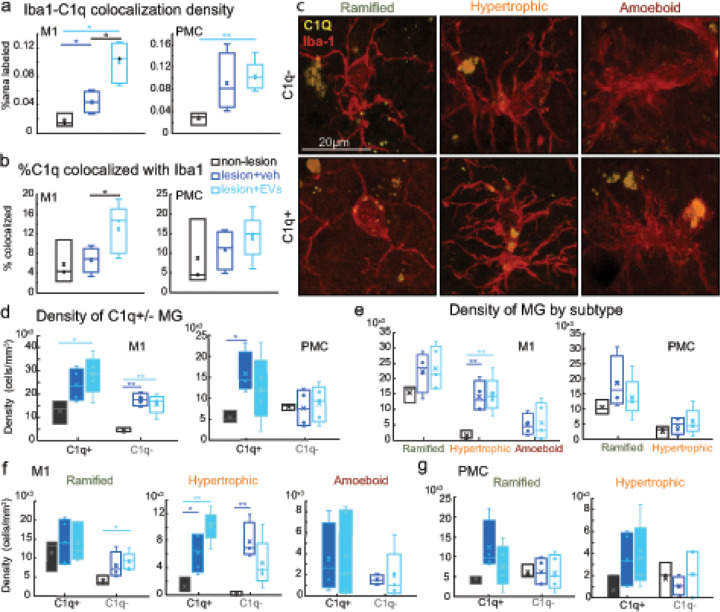
C1q expression in different microglia phenotypes. **a** The density (% area label) of Iba1+ colocalized with C1q+ puncta (Fisher’s LSD *post hoc*, M1: con. vs. veh, p=0.046; con. vs. EV, p=0.003; veh vs. EV, p=0.009); PMC: con. vs. EV p=0.004. Non-lesion control: n=3. Veh group: n=4. EV group: n=5). **b** Higher fraction of VGLUT2 colocalized with microglia (Iba1) in EV than in the veh. (Fisher’s LSD *post hoc*, veh. vs. EV: p=0.05. Non-lesion control: n=3. Veh group: n=4. EV group: n=5). **c**Representative images of dual labeling of microglia (red) and C1q (yellow), with examples of different microglia morphologies dual labeled with C1q. **d**Cell densities of total C1q+ vs C1q- microglia. In M1, C1q- microglia: greater density in lesion than control (*t*-test, con. vs. veh: p<0.001; con. vs. EV: p=0.004); EV but not veh had greater C1q+ microglia density than control (*t*-test, con. vs. veh: p=0.07; con. vs. EV: p=0.03). In PMC, greater density of C1q+ microglia in veh than control group (*t*-test, con. vs. veh: p=0.02). **e** In M1, hypertrophic microglia: greater density in lesion compared to control group (*t*-test, con. vs. veh: p=0.01; con. vs. EV: p=0.008). **f** In M1, the EV group showed higher density of C1q- ramified microglia as compared to the control group (*t*-test, con. vs. EV: p=0.01). Both groups with lesion had higher density of hypertrophic C1q+ microglia as compared to the controls (*t*-test, con. vs. veh: p=0.047; con. vs. EV: p<0.001). However, only veh had higher density of hypertrophic C1q- microglia as compared to the controls (*t*-test, con. vs. veh: p=0.005). **g** In PMC, no between-group difference was found in the density of ramified or hypertrophic microglia. (**d-g** non-lesion control: n=3. Veh group: n=4. EV group: n=5)

While we found a lesion effect on C1q tagging of VGLUT2 + excitatory boutons, we did not find EV treatment effects on C1q expression and VGLUT2 tagging. Since microglia produce and store C1q [15], as well as phagocytose C1q tagged debris, we therefore assessed the Iba-1-C1q colocalization. We found a lesion-related increase in the total density (percent area) of Iba-1-C1q colocalization in M1 in both treatment groups (Fig. 7a, Fisher’s LSD *post hoc*, M1: con. vs. veh, p < 0.05; con. vs. EV, p < 0.01). In PMC, only the EV group exhibited a significant increase in Iba-1-C1q colocalization density compared to non-lesion control (Fig. 7a, Fisher’s LSD *post hoc*, PMC: con. vs. EV, p < 0.01). Further a treatment effect was found in M1, with EV treated monkeys having significantly greater fraction of C1q puncta colocalized with Iba-1 compared to vehicle group (Fig. 7b, M1: Fisher’s LSD *post hoc*, veh. vs. EV, p < 0.05).

Given the findings from our previous study that EV promoted a morphological shift from inflammatory to homeostatic ramified microglia [13], we therefore assessed whether this increased C1q + expression in microglia was associated with specific microglial morphologies that are thought to reflect distinct immune activation states [22]. Ramified microglia are characterized by small round somata, thin and highly branched processes, thought to be in a surveilling homeostatic state. Upon immune activation, microglia transition to an amoeboid state, which have enlarged somata, with few short and thick processes and are thought to be in a phagocytic state. A transitional state between ramified and amoeboid states are hypertrophic microglia that have intermediate thick process, more polarized or ovoid intermediate sized somata, and branching can be extensive or not depending on state of transition. Molecular identification of microglia specifically in the ‘polarized’ hypertrophic states suggest distinct sub-populations that have either downstream anti-inflammatory or pro-inflammatory effects [19].

We classified Iba-1 + microglia based on their C1q expression (C1q + vs C1q-) and their morphologies– ramified, hypertrophic, or amoeboid in perilesional M1 and PMC (Figs. 7c). In both veh and EV lesion groups, there was a greater density of C1q- microglia as compared to the non-lesion control group in M1 (Fig. 7d, C1q-: con. vs. veh, p < 0.01; con. vs. EV, p < 0.01). However, we found regional and treatment related differences specifically in the density of C1q + microglia. Specifically, consistent with particle analyses data, EV but not veh group had significantly greater density of C1q + microglia in M1 as compared to the non-lesion controls (Fig. 7d, t-test, M1 C1q+: con. vs. EV, p < 0.05). Conversely, in PMC, veh but not EV group had a higher density in C1q + microglia compared to the controls (Fig. 7d, t-test, PMC C1q+: con. vs. veh, p < 0.05).

Assessments of total Iba-1 + microglia by morphological subtype revealed a lesion-related increase in the density of hypertrophic microglia of both veh and EV groups only in M1 (Fig. 7e, t-test, M1 Hypertrophic: con. vs. veh, p < 0.01; con. vs. EV, p < 0.01). This lesion-related increase in hypertrophic microglia in M1 is due to an increase in the density of C1q + hypertrophic microglia in the EV monkeys, while an increase in both C1q + and C1q- hypertrophic microglia in the veh monkeys (Fig. 7f, Density of Hyper C1q + *t*-test: con. vs. veh, p < 0.05; con. vs. EV, p < 0.01; C1q-: con. vs. veh, p < 0.01). Further in M1, the EV group but not veh group had a higher density of C1q- ramified microglia than control group (Fig. 7f, Density of Rami C1q- *t-* test: con. vs. EV, p < 0.05). No significant between-group differences in the densities of microglia subtypes were found in PMC (Fig. 7g).

**Figure 8:**
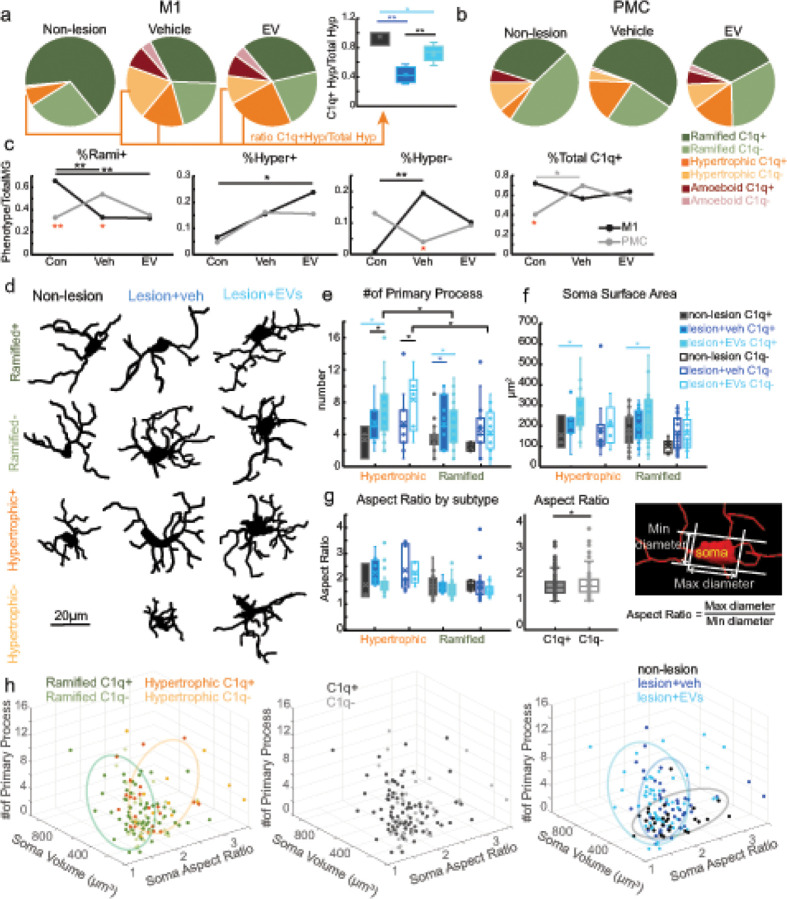
The expression of different microglia phenotypes in M1 and PMC. **a** Pie charts of %microglia by morphology and C1q expression in M1. Right inset shows the ratio of Hyper+ to the total Hyper microglia in each group (*t*-test, con. vs. veh: p=0.001; con. vs. EV: p=0.03; veh. vs. EV: p=0.006). **b** In PMC (Rami-veh < con.: p=0.05; EV vs con.: p=0.49). **c** Relative proportion of Rami+, Hyper+, Rami-, and Total C1q+ normalized to the total number of microglia counted for each case. Significant group*area interaction for %Rami+ (p=0.008), %Hyper- (p=0.02), and %Total C1q+ (p=0.04). A main effect of group for %Hyper+ (p=0.01). Significant between-group differences per area: M1 (%Rami+ veh < con, p=0.006; EV vs con, p=0.004; %Hyper+, EV > con, p=0.01; %Hyper- veh. > con. p=0.007). PMC (%C1q+ veh > con, p=0.02). Significant between-area differences per group: Control (%Rami+ M1 > PMC, p=0.009; and %C1q+ M1 > PMC, p=0.02). Vehicle (%Rami+: PMC > M1, p=0.05; but %Hyper-: M1 > PMC, p=0.01). Non-lesion control: n=3. Veh group: n=4. EV group: n=5. **d**Representative microglia reconstructions. **e-g** Morphological parameters (non-lesion control: n=36 cells from 1 monkey. veh group: n=58 cells from 1 monkey. EV group: n=71 cells from 1 monkey): **e** Number of primary process (three-way ANOVA, main effect ‘morphology’: p=0.02; ‘group’: p=0.01). Hyper+ (Fisher’s LSD *post-hoc* EV > con, p=0.03; EV > veh, p = 0.05); Hyper- (EV > veh, p = 0.02); Rami+ (con < veh, p=0.03; con < EV, p=0.05); EV group (Hyper+ > Rami+: p=0.004; Hyper- > Rami-: p<0.001). **f** Microglia soma surface area (three-way ANOVA, main effect ‘group’: p=0.006; ‘C1q+/−’: p=0.004). Hyper+: EV > con., p=0.01; Rami+: EV > con, p=0.04. **g** Soma aspect ratio (three-way ANOVA, main effect ‘C1q+/−’: p=0.03). **h** 3D scatter plot of morphological parameters. Annotations based on morphology, C1q expression, and experimental group

We then assessed whether the density and relative proportion of each microglia phenotype based on morphology and C1q expression were altered by lesion and treatment. Importantly, two-way ANOVA of experimental group*area showed that lesion-related shifts in the distribution of microglia subtypes by C1q expression and morphology were region-dependent. Between-group comparison showed that in M1 but not PMC there was a lesion-related increase in density of total and C1q+ (Hyper+) hypertrophic microglia (Fig. 7e: Fisher’s LSD *post hoc*, M1 Hyper con. vs. veh.: p < 0.001; con. vs. EV: p < 0.001; Fig. 7f, Fisher’s LSD *post hoc*, M1 Hyper + con. vs. veh.: p < 0.05; con. vs. EV: p < 0.001). This group*area interaction effect was also found in the proportions of microglia subtypes, indicating that lesion differentially shifted the relative proportions of microglia subtypes in a region-dependent matter (Fig. 8a–c, two-way ANOVA, area*group interaction, %Rami+: p < 0.01; %Hyper-: p < 0.05; %C1q+: p < 0.05). Specifically, M1 but not PMC exhibited lesion-related reduction of % C1q + ramified (Rami+) microglia (Fig. 8c, Fisher’s LSD *post hoc*, M1%Rami + con. vs. veh.: p < 0.01; con. vs. EV: p < 0.01), and lesion-related increase in % C1q- hypertrophic (Hyper-) microglia (Fig. 8c, Fisher’s LSD *post hoc*, M1%Hyper- con. vs. veh.: p < 0.01). Interestingly, there was an EV treatment effect on the proportion of C1q + vs C1q-hypertrophic microglia, which also varied based on regions. Specifically, two-way ANOVA results demonstrated that the EV treated group but not the veh group had a significantly higher proportion of hypertrophic C1q + microglia in M1 as compared to the controls (Fig. 8c, Fisher’s LSD *post hoc*, %Hyper + M1: con. vs. EV: p < 0.05). In contrast, there was a greater proportion of hypertrophic C1q- microglia in the M1 of the veh group compared to non-lesion controls (Fig. 8c, Fisher’s LSD *post hoc*, %Hyper-: con. vs. veh, p < 0.05).

**Figure 9:**
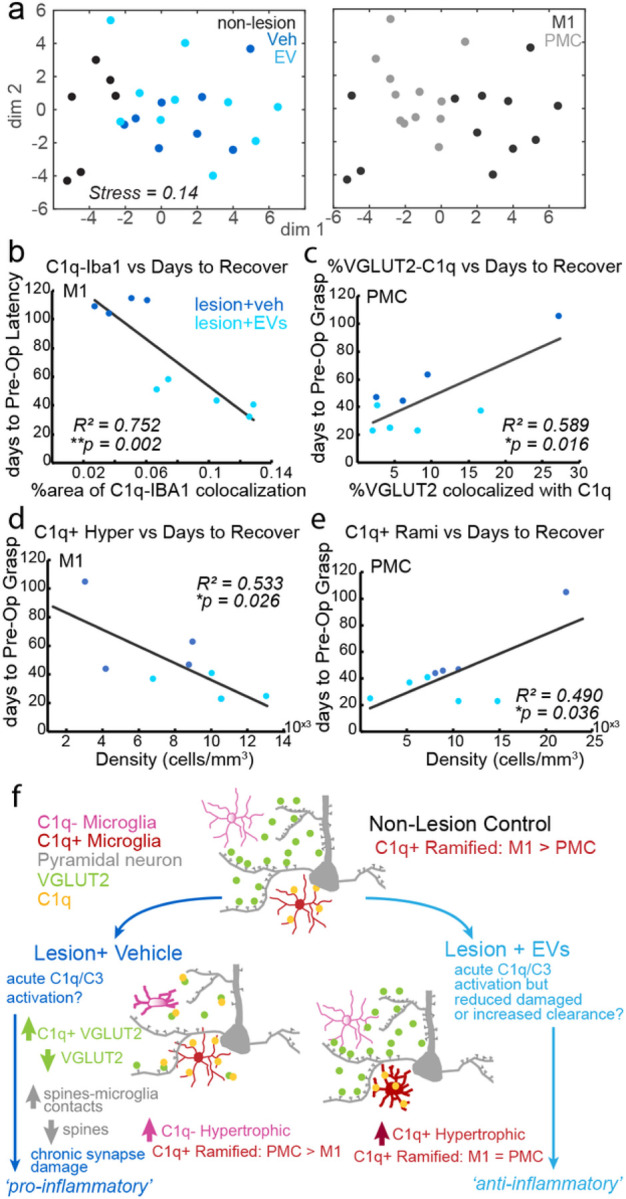
Relationship of synaptic and microglia properties to behavior outcome features. **a** NMDS plot showing clustering of cases, annotated by experimental group (left) and cortical area (right), based on 21 synaptic and microglia outcome measures (%area VGLUT1, VGLUT2, VGAT, GLUR2/3, GABAA alpha1, GABAB R2; % of VGLUT1, VGLUT2 or VGAT with Iba1; % of IBA1 with VGLUT1, VGLUT2 or VGAT; % area C1q; % of VGLUT2 with C1q; % C1q with VGLUT2; cell densities of Ramified, Hypertrophic, Amoeboid C1q+ and C1q- microglia). The proximity of points indicates the relative similarity-based pair-wise correlation of these multiple variables. **b-e** Significant linear correlations between synaptic-microglial measures and behavioral outcome measures: **b** Increased density of C1q and Iba1 colocalization in M1 correlated with faster recovery time (less days to return to pre-operative latency to retrieve food reward; *R*^*2*^ = 0.752, p=0.002). **c** Increased fraction of VGLUT2 colocalized with C1q in PMC correlated with slower recovery time (more days return to preoperative grasp pattern; *R*^*2*^ = 0.589, p=0.016). **d** Greater expression of C1q+ hypertrophic microglia in M1 was correlated with faster recovery time (*R*^*2*^ = 0.533, p=0.026). **d** Greater expression of C1q+ ramified microglia in PMC was associated with slower recovery time (*R*^*2*^ = 0.490, p=0.036). **f** A schematic showing summary of findings and proposed model of the lesion and EV treatment effects on microglial-synapse modulation and C1q signaling pathways. Cortical lesion in M1 induces acute damaged in neuronal structures that triggers an acute increase in C1q+ signaling cascade to initiate phagocytotic clearance. The veh group had accumulation of further damage and downstream C1q pathway related proteins that sustains a chronic pro-inflammatory response (C1q- hypertrophic microglia). The EV treatment upregulated C1q+ mediated clearance of debris and facilitated an early shift to the anti-inflammatory C1q+ hypertrophic microglia phenotype that persisted in the chronic stages, thereby supporting functional recovery

Given that we found between-group differences in hypertrophic microglia, we assessed the ratio of C1q + hypertrophic microglia to total hypertrophic microglia in M1 for all three groups (Fig. 8a, box-whisker plots). Our results showed that the in M1 of non-lesion control animals almost all (94%) hypertrophic microglia were C1q+, and this proportion was greater compared to the groups with lesion (Fig. 8a, C1q + Hyper/Total Hyper *t*-test: con. vs. veh, p < 0.01; con vs. EV, p < 0.05). EV treatment attenuated this lesion- related proportional decrease in C1q + hypertrophic microglia. In the vehicle group, about 40% of hypertrophic microglia were C1q+; in the EV group, this proportion was significantly greater, with about 70% of hypertrophic microglia expressing C1q+ (Fig. 9a, C1q + Hyper/Total Hyper *t*-test: veh vs. EV, p < 0.01).

### 3.10 Lesion and EV treatment affects region-specific expression of microglia phenotypes.

The data above indicated that EV treatment affected specifically hypertrophic C1q + expression in M1. However, EV treatment seemed to also affect the lesion-related shifts in between-area differences in distribution of microglial types in M1 and PMC. Notably, in non-lesion control, a between-area difference was found in the density and proportion of C1q + ramified and hypertrophic microglia. A two-way ANOVA analysis revealed a main effect of area on the density of different microglia subtypes (Fig. 7d–g, two-way ANOVA, area main effect, Rami+: p < 0.05; Hyper+: p < 0.01; Hyper-: p < 0.05; TotalRami: p < 0.05; TotalHyper: p < 0.01; TotalC1q+: p < 0.01; TotalC1q-: p < 0.01), indicating that microglia phenotypes were expressed differently in M1 and PMC. In M1, majority of both ramified and hypertrophic microglia were C1q+. In PMC, majority of hypertrophic microglia were C1q- and a more equal distribution of C1q + and C1q- ramified microglia was found (Fig. 8a–b). Compared to PMC, M1 had greater density of Rami + but not Rami- microglia, and greater density of both Hyper + and Hyper- microglia (Fig. 7f–g). Consistently, the proportion of Rami + microglia is greater in M1 than in PMC (Fig. 8c, t-test, con. %Rami + M1 vs. PMC: p < 0.01). Lesion in the vehicle group shifts this distribution to the opposite pattern, with %Rami + microglia lower in M1 than in PMC (Fig. 8c, Fisher’s LSD *post hoc*, %Rami + veh., M1 vs. PMC: p < 0.05). Lesion in the vehicle group also caused relative increase in %Hyper- microglia in M1 compared to PMC, that did not differ significantly in the non-lesion control brains (Fig. 8c, Fisher’s LSD *post hoc*, %Hyper- con., M1 vs. PMC: p = 0.07; veh., M1 vs. PMC: p < 0.05). Interestingly, EV treatment seemed to dampen this lesion-related decrease in %Rami + and increase in % Hyper- in M1 relative to PMC (Fig. 8c).

In summary, between-group within area comparisons showed that in M1, lesion proportionally decreased ramified microglia, but increased hypertrophic microglia compared to non-lesion control (Fig. 7&8). The opposite pattern was seen in PMC, where lesion shifted to a proportional increase of C1q + ramified microglia but decrease in C1q- hypertrophic microglia (Fig. 8a–c). Further, there are between-area differences in the normative distribution of these microglia. Our results also demonstrated that EV treatment regulated the post-injury region-dependent expression of microglia subtypes by reversing or attenuating the lesion caused changes.

### 3.11 Microglia morphological features are dependent on experimental group and C1q expression

Overall, our results indicated that lesion and EV treatment facilitated a specific phenotypic shift in C1q + vs C1q- hypertrophic, and to a lesser extent ramified, microglia in a region dependent manner. We thus further assessed whether these C1q + vs C1q- hypertrophic and ramified microglia represented distinct subclasses with unique morphologic features. Using 3D reconstruction methods, we quantified morphological features of individual microglia in M1 and assessed the independent and interactive effects of morphology*C1q expression*experimental group (Fig. 8d–g). We were not able to report reconstruction data for C1q- hypertrophic microglia in control animals since this subpopulation was very rare in this group. There were significant main effects of group and morphology on the number of primary processes (Fig. 8e: three-way ANOVA, group/morphology main effect, p < 0.05). Specifically, microglia in both groups with lesion had a greater number of primary processes as compared to the controls. Ramified C1q + microglia in both groups with lesion showed a greater number of primary processes as compared to the controls (Fig. 8e, Fisher’s LSD *post hoc*, Rami C1q+: con. vs. veh: p < 0.05; con. vs. EV: p < 0.05). In addition, there was a treatment effect on hypertrophic C1q+/− microglia, which exhibited a greater number of processes in the EV-treated group compared to those in vehicle and control groups (Fig. 8e, Fisher’s LSD *post hoc*, Hyper C1q+: con. vs. EV: p < 0.05; veh. vs. EV: p < 0.05; Hyper C1q-: veh. vs. EV: p < 0.05). For all C1q+/− microglia in the EV group, the hypertrophic microglia had greater numbers of primary processes as compared to the ramified cells (Fig. 8e, Fisher’s LSD *post hoc*, C1q + Hyper. vs. Rami: p < 0.05; C1q- Hyper. vs. Rami: p < 0.05). These results demonstrated that regardless of C1q expression, microglia from the EV-treated group was characterized by a greater number of primary processes, consistent with our previous work [13].

Significant main effects of C1q expression (+/−) and group were found for features of ramified and hypertrophic microglia somata (Fig. 8f: three-way ANOVA, group/C1q expression main effect, p < 0.01). Both hypertrophic and ramified C1q + microglia in EV group but not the veh group had greater surface area of their soma as compared to the non-lesion control group (Fig. 8f, Fisher’s LSD *post hoc*, C1q + Hyper. con. vs. EV: p < 0.05; C1q + Rami. con. vs. EV: p < 0.05). In addition, a significant main effect of C1q expression was found for cell body aspect ratio, which was calculated as the maximum diameter of the microglial somata divided by the minimum diameter (Fig. 8g, three-way ANOVA, main effect, p < 0.05). Aspect ratio can be a measure of microglia polarization and immune activation [30]. Cell bodies of C1q + microglia (ramified or hypertrophic) exhibited a smaller aspect ratio (rounder) compared to the C1q- microglia which had a more elongated, oval shape. These results showed that C1q expression on microglia was associated with rounder somata with larger surface areas.

We then assessed whether these unique set of morphological features will reveal distinct clusters of microglia subtypes. For each microglia reconstructed, we plotted a 3D scatter plot of primary process, soma volume, and soma aspect ratio, and annotated the plots based on morphology and C1q expression, cortical region and experimental group (Fig. 8h). 3D scatter plot revealed some clustering of microglia based mainly on morphology and not C1q expression (Fig. 8h). Specifically, ramified microglia were associated with smaller soma volume and rounder somata shape (smaller aspect ratio) as compared to the hypertrophic microglia (Fig. 8h, left panel). Our clustering analysis also showed that microglia in the three groups exhibited different morphologies (Fig. 8h, right panel). The non-lesion controls tend to have smaller soma with a broader range of aspect ratio and less primary processes. Microglia in the EV group was shown to have larger soma and more processes, whereas the vehicle group had intermediate soma volume but a higher aspect ratio indicating an oval somata shape (Fig. 8h right panel). In contrast to morphological categories and experimental group, no clustering of microglia based on these 3 morphological features was found between regions (Fig. 8h, middle panel).

We then determined the combined effects of synaptic and microglia outcome measures on the relative (dis)similarities of areas and experimental groups in this study. Thus, we performed non-metric multidimensional reduction and clustering of individual PMC and M1 tissue from each case, based on a distance proximity matrix derived from pair-wise correlation of 21 synaptic and microglia outcome measures (per case: % area VGLUT1, VGLUT2, VGAT, GLUR2/3, GABA_a_ *α*1, GABA_b_ R2; % of VGLUT1, VGLUT2 or VGAT with Iba-1; % of Iba-1 with VGLUT1, VGLUT2 or VGAT; % area C1q; % of VGLUT2 with C1q; % C1q with VGLUT2; cell densities of Ramified, Hypertrophic, Amoeboid C1q + and C1q- microglia). Our analyses note that there is a strong separation between the control from the lesion group. Further, within each experimental group, M1 vs PMC are separated. This regional separation is most prominent within the non-lesion control group, highlighting the normative diversity between cortical areas with regards to synaptic and microglial features [17, 31].

### 3.11 C1q-VGLUT2 tagging correlated with functional recovery after cortical injury

Our previous study [12] has reported that compared with vehicle-treated monkeys, EV-treated monkeys exhibited enhanced recovery of fine motor function of the hand, evidenced by the fewer number of days to return to pre-operative hand grasp pattern and latency to retrieve food reward. Using the functional recovery data from Moore et al. (2019) and Pessina et al. (2019) [12, 32], we used linear regression analyses to determine whether the cellular data reported above were associated with behavioral measures of motor recovery (number of days to return to pre-operative latency and grasp pattern). Our results showed that increased C1q-Iba-1 colocalization in M1—specifically increased density of C1q + hypertrophic microglia—was associated with a more rapid recovery rate (fewer days to return to pre-operative latency; Fig. 9b, *R*^*2*^ = 0.752, *p* < 0.01; Fig. 9d, *R*^*2*^ = 0.533, *p* < 0.05). In PMC, we found increased portion of VGLUT2 tagged by C1q in PMC was associated with a slower recovery rate (more days to return to preoperative grasp pattern; Fig. 9c, *R*^*2*^ = 0.589, *p* < 0.05). Increased density of C1q + ramified microglia in PMC was also associated with a slower recovery rate (more days to return to preoperative grasp pattern, Fig. 9e, *R*^*2*^ = 0.49, *p* < 0.05). Overall, our results indicated that the C1q + hypertrophic microglial expression in perilesional M1 was beneficial for the recovery of fine motor function. In contrast, C1q tagging on VGLUT2 + synapse in PMC was detrimental for recovery, indicated by the slower recovery rate.

## 4. DISCUSSION

Previous studies have demonstrated the effects of EVs on shifting microglial morphological phenotypes [13] and ameliorating synaptic imbalance in cortical and spinal motor circuits [14, 33], to support recovery of motor function after cortical injury in primary motor cortex (M1). The current study provides a mechanistic link between these previous data, showing complementary effects of EV treatment on synaptic marker and microglial expression, and their structural and molecular relationships. As summarized in Fig. 9f, both lesion and EV treatment modify microglial-synapse relationships and microglial phenotypic expression of the complement pathway initiator protein, C1q, in a region-dependent manner. Compared to vehicle, EV treatment was associated with increased expression of C1q + hypertrophic microglia and decreased expression of C1q- hypertrophic microglia in M1, but decreased expression of C1q + ramified microglia in PMC (Fig. 9f). These data point to the role of EV-mediated regulation of region and circuit-specific synaptic plasticity to support recovery of motor function after cortical injury.

### 4.1 Differential effects of injury on excitatory VGLUT1 and VGLUT2 terminals suggest pathway-specific mechanisms for plasticity

Cortical injury leads to neuronal hyperexcitability, excitotoxicity, and disruption of synaptic transmission in perilesional cortex [3, 14]. Glutamate, the major excitatory neurotransmitter in the central nervous system, is stored and transported into synaptic vesicles by presynaptic vesicular glutamate transporters (VGLUTs) that have isoforms differentially expressed across distinct neuronal pathways [34]. In the adult brain, VGLUT1 is expressed mainly by cortico-cortical axons, while VGLUT2 is mainly expressed in subcortical glutamatergic neurons, predominantly in thalamocortical axons [35]. The current results showed significant reduction of VGLUT2 in both groups with lesion compared to non-lesion controls, while no significant difference was found in VGLUT1. These results suggest that either axon terminals expressing VGLUT2 + may be selectively vulnerable to injury compared to VGLUT1+, or that VGLUT1 + connections have a greater degree of plasticity after injury. This is consistent with literature suggesting that axon terminals expressing VGLUT1 such as those in the hippocampus exhibit higher potential for plasticity than those expressing VGLUT2, such as the climbing fibers in the cerebellum [36]. Future experiments will be important to clarify the molecular pathways underlying the differential regulation of VGLUT1 and VGLUT2 expression after injury.

### 4.2 EV treatment ameliorated injury-related dysregulation of glutamate AMPA receptor subunit gene expression in PMC

Glutamatergic AMPA receptor composition is an important determinant of synaptic strength and plasticity [3]. The GLUR2 AMPAR subunit, in particular, controls calcium permeability, thereby affecting AMPARs traffi cking, and spine growth [37, 38]. In the present study, lesion-related reduction of GLUR2/3 receptors, which is dampened in the EV treated group. However, mRNA expression *GRIA2*, the gene for GLUR2 AMPA receptor subunit, showed the opposite trend; the lesion-related increase in *GRIA2* mRNA was downregulated and normalized by EV. The opposite effects of lesion on GLUR2/3 protein and *GRIA2* mRNA may be due to numerous factors. First, the *GRIA2* mRNA is related to GLUR2 protein translation, but not GLUR3 receptors. Second, the mRNA-protein discrepancy suggests differences in transcriptional and post-translational regulation of plasticity in response to lesion and lesion-induced hyperexcitability. Previous work in rodents has shown that increased protein expression of GLUR2 promotes dendritic spines formation and enlargement in rat hippocampal neurons [39]. Upregulation of *GRIA2* mRNA and concomitant downregulation of *GRIA1* (GLUR1) mRNA was found in response to pharmacologically-induced hyperexcitability in neuronal cultures [40]. The current data is consistent with our previous findings showing lesion-induced hyperexcitability, accompanied by excitatory synapse loss at the electrophysiological and structural level in vPMC [14]. Thus, it is possible that lesion-related *GRIA2* mRNA upregulation resulted from increased hyperexcitability, but the downstream mechanisms for GLUR2/3 subunit protein synthesis, traffi cking and insertion were impaired, thereby not allowing for spine and synapse growth after injury. Interestingly, the current data suggest that this potential impairment in post- translational regulation is apparent only in vehicle monkeys, where upregulated GLUR2 mRNA was found together with downregulated GLUR2/3 protein expression. In EV-treated monkeys, GLUR2 mRNA and GLUR2/3 protein levels, which showed the opposite trend from vehicle monkeys, were normalized closer to baseline non-lesion control. This is consistent with our previous work demonstrating that the lesion-related decrease in excitatory postsynaptic current frequencies and spine loss were ameliorated by EVs [14]. Together, these data suggest that EV-mediated dampening of chronic hyperexcitability in perilesional neurons, can be associated with preventing aberrant plasticity and maintaining glutamatergic synapse growth.

### 4.3 Differential effects of injury on distinct inhibitory neurotransmitter receptor expression

In addition to changes in excitatory neurotransmission, phasic and tonic inhibitory GABAergic transmission, conferred by distinct ionotropic GABA_a_ and metabotropic GABA_b_ receptor subunits [41], also play a complex role in recovery after injury [42, 43]. Here, we found in both M1 and PMC, a lesion-related reduction in the density of GABA_a_ *α*1, a subunit localized on synaptic membranes mediating fast phasic inhibitory currents [41]. Further, in our qPCR results, we found a lesion-related increase in gene expression of GABA_a_ ∂ (*GABRD*) and GABA_b_ R2 (*GABBR2*) subunits, known to mediate tonic inhibitory currents that control overall cell excitability [3, 25, 44]. The current results are consistent with previous studies in rodent and *in vitro* models showing that injury results in increased tonic inhibition to prevent excitotoxicity during the acute recovery period [42, 43]. However re-establishing phasic inhibitory transmission is needed to support reorganization [42, 43]. While the current data did not show a treatment effect with regards to GABA receptors, our previous study showed that EV treatment was associated with a specific increase in distal apical inhibitory synapses and task-related immediate early gene activation of dendritic-targeting inhibitory interneurons that support recovery of motor function [14]. Thus, cell-type and compartment specific changes in GABAergic receptor expression across recovery would be important to assess in future work.

### 4.4 EV treatment upregulated the anti-inflammatory C1q +hypertrophic microglia in M1

While activity-dependent, neuronal mechanisms of synaptic plasticity have been well studied, it is only recently that the role of microglia and neuro-immune signaling have been investigated [45, 46]. In the healthy brain, microglia can regulate synaptic turn-over through phagocytosis of synapses for pruning, or releasing trophic factors to promote synapse growth [45, 47]. After injury, microglia, as the resident macrophages of the brain, are stimulated to mediate clearance of damaged synapses [48]. A crucial part of this microglial mediated neuro-immune signaling is the complement system [15, 20]. During periods of active synaptic pruning or after injury, the initiating protein of the classical complement cascade, C1q, is produced by microglia to tag excess or damaged synapses. C1q tagging then triggers downstream deposition and activation of complement effector molecules, such as C3 receptors, which would in turn lead to microglial synapse phagocytosis [28, 29, 49]. In the present study, the vehicle group showed a lesion-related elevation in C1q tagging of VGLUT2 + axon terminals (C1q+/VGLUT2 + colocalization), coupled with decreased VGLUT2 + density, suggesting greater synapse damage and loss [14]. However, in the EV group, this C1q-synapse tagging was dampened, and coupled with a greater expression of C1 + hypertrophic microglia in perilesional M1 (Fig. 9f). After cortical injury, microglia can be ‘immune-activated’ to exhibit distinct macrophage-like phenotypes polarized towards either pro- and anti-inflammatory functions [50–52]. Synthesis of C1q within anti-inflammatory (M2 macrophage-like) microglia has been shown to be acutely upregulated after cortical injury, playing a protective role, to promote the clearance of apoptotic cells and secretion of anti-inflammatory cytokines, and suppress production of pro-inflammatory cytokines [6, 8] [29, 53]. Thus, EV treatment mitigated a sustained chronic pro-inflammatory state, evidenced by increased expression of the protective and debris-clearing C1 + hypertrophic microglia in perilesional cortex 12 weeks post-injury. Indeed, here we show that greater expression of C1q + hypertrophic microglia in perilesional M1 was associated with more rapid recovery of function. Interestingly, we found that the vehicle group exhibited a decrease in the proportion of C1q + but an increase in C1q- hypertrophic microglia in perilesional M1, compared to EV and control groups (Fig. 9f). Thus, these C1q- hypertrophic microglia represents a distinct population, likely belonging to the pro-inflammatory subclass [50, 51], which persisted in the vehicle monkeys. These pro-inflammatory microglia can exacerbate neurotoxicity by releasing pro-inflammatory cytokines including TNF-*α*, IL-6, and IL-1β [4, 54].

In a mouse model of Alzheimer’s disease [55], it was found that early in the disease, C1q ‘primed’ microglia are anti-inflammatory and protective. However, after C1q release and tagging, the downstream molecular targets of C1q can either promote anti-inflammatory (via C3b receptor activation) or exacerbate chronic pro-inflammatory (via C3a or C5 receptor activation) signaling [29]. Future studies to assess the temporal progression of distinct C1q effectors will be important to further understand the role of EVs in modulating the complement system to support recovery after cortical injury. Nevertheless, the current findings together with our previous data [13] support the role of EV treatment in inducing an early shift from pro-inflammatory to anti-inflammatory microglia in perilesional cortex, preventing chronic inflammation and damage after cortical injury.

### 4.5 Region-dependent effects of injury and treatment in modulating microglial phagocytosis.

The current findings revealed novel region-specific expression of microglial phenotypes that are differentially modulated by injury and EV treatment. In the non-injured brain, ramified homeostatic microglia predominated. However, there were baseline regional differences in the proportion of C1q + vs C1q- ramified microglia, with M1 showing a significantly greater proportion of C1q + microglia than PMC. These data are consistent with previous work in the rodent and human brain, highlighting diversity across cortical areas with regards to microglial subpopulations [56–59]. Further, the present findings suggest innate differences in microglial/C1q dependent synapse turnover between two cortical motor areas, with synapses in M1 likely subject to greater synaptic pruning via the upregulated C1q + ramified microglia compared to PMC [46, 60]. Interestingly, the lesion alone resulted in a reversal of the M1 vs PMC gradient in C1q + ramified microglia; However, EV treatment mitigated this lesion-related regional shift. Indeed, while increasing expression of C1q + hypertrophic microglia in M1 was associated with a more rapid recovery rate, increased C1q-synapse (VGLUT2) tagging and C1q + ramified microglia in PMC were associated with a prolonged recovery. These results suggest that microglia phagocytosis can be beneficial in perilesional M1, which likely reflect the neuroprotective effects of clearance of damaged tissue necessary to promote tissue repair and re-establish homeostasis [4]. However, chronic and excessive phagocytosis of synapses in PMC can exacerbate neuronal cell death and loss of connections [5, 45, 46, 61] that can be detrimental to recovery.

## 5 Conclusion

We have elucidated the potential microglia-synapse and complement C1q-related mechanisms of how MSC-EVs treatment can enhance recovery in a monkey model of cortical injury. Cortical lesion results in reduced expression of excitatory and inhibitory synaptic markers in perilesional M1 and PMC, consistent with the functional deficits in excitatory and inhibitory synaptic transmission shown in monkeys [14] and rodents [42]. The current data suggest that EV treatment facilitated synaptic plasticity by regulating microglial activity in a region-dependent manner—enhancing anti-inflammatory C1q + hypertrophic microglia expression in perilesional M1 for clearance of acute damage, and thereby preventing excessive synaptic loss in PMC and chronic synaptic dysfunction. These mechanisms may act to preserve synaptic cortical motor networks and balanced normative M1/PMC synaptic connectivity to support functional recovery after injury [14, 62–64]. However, it still remains unclear whether EVs act on microglia directly or indirectly via upstream signaling pathways [4] or via suppressing pro-inflammatory complementary system markers downstream to C1q. Our findings form the basis of future studies to dissect the molecular effects of MSC-EVs treatment on the complement cascade crucial for recovery after cortical injury, and extend their therapeutic application for acute and chronic neurodegenerative diseases.

## Data Availability

The raw data that support the findings in the present study are available from the corresponding author upon request.

## Abbreviations

MSC-EVs: Mesenchymal stem cell-derived extracellular vesicles
M1: Primary motor cortex
PMC: Premotor cortex
VGLUT: Vesicular glutamate transporter
GLUR: Glutamate receptor
AMPA: α-amino-3-hydroxy-5-methyl-4-isoxazolepropionic acid
GABA: γ-aminobutyric acid
VGAT: Vesicular GABAergic transporters
Iba-1: Ionized calcium binding adaptor molecule 1
P2RY12: Purinergic receptor P2Y, G-protein coupled, 12
PB: Phosphate buffer
BSA: Bovine serum albumin
NDS: Normal donkey serum
R+/Rami +: Ramified microglia with C1q expression
R-/Rami-: Ramified microglia without C1q expression
H+/Hyper +: Hypertrophic microglia with C1q expression
H-/Hyper-: Hypertrophic microglia without C1q expression
A+/Ame +: Amoeboid microglia with C1q expression
A-/Ame-1: Amoeboid microglia without C1q expression
